# The IUPHAR Guide to Immunopharmacology: connecting immunology and pharmacology

**DOI:** 10.1111/imm.13175

**Published:** 2020-03-02

**Authors:** Simon D. Harding, Elena Faccenda, Christopher Southan, Adam J. Pawson, Pasquale Maffia, Stephen P. H. Alexander, Anthony P. Davenport, Doriano Fabbro, Francesca Levi‐Schaffer, Michael Spedding, Jamie A. Davies

**Affiliations:** ^1^ Deanery of Biomedical Sciences University of Edinburgh Edinburgh UK; ^2^ Centre for Immunobiology Institute of Infection, Immunity and Inflammation College of Medical, Veterinary and Life Sciences University of Glasgow Glasgow UK; ^3^ Institute of Cardiovascular and Medical Sciences College of Medical, Veterinary and Life Sciences University of Glasgow Glasgow UK; ^4^ Department of Pharmacy University of Naples Federico II Naples Italy; ^5^ School of Life Sciences University of Nottingham Medical School Nottingham UK; ^6^ Experimental Medicine and Immunotherapeutics University of Cambridge Cambridge UK; ^7^ Cellestia Biotech SA Basel Switzerland; ^8^ TargImmune Therapeutics AG Basel Switzerland; ^9^ Institute for Drug Research The Hebrew University of Jerusalem Jerusalem Israel; ^10^ Spedding Research Solutions SAS Le Vésinet France; ^11^ Present address: TW2Informatics Ltd Göteborg 42166 Sweden

**Keywords:** database, immune‐therapeutics, immunopharmacology, pharmacology

## Abstract

Given the critical role that the immune system plays in a multitude of diseases, having a clear understanding of the pharmacology of the immune system is crucial to new drug discovery and development. Here we describe the International Union of Basic and Clinical Pharmacology (IUPHAR) Guide to Immunopharmacology (GtoImmuPdb), which connects expert‐curated pharmacology with key immunological concepts and aims to put pharmacological data into the hands of immunologists. In the pursuit of new therapeutics, pharmacological databases are a vital resource to researchers through providing accurate information on the fundamental science underlying drug action. This extension to the existing IUPHAR/British Pharmacological Society Guide to Pharmacology supports research into the development of drugs targeted at modulating immune, inflammatory or infectious components of disease. To provide a deeper context for how the resource can support research we show data in GtoImmuPdb relating to a case study on the targeting of vascular inflammation.

## Graphical abstract

The International Union of Basic and Clinical Pharmacology (IUPHAR) Guide to Immunopharmacology (GtoImmuPdb) connects expert‐curated pharmacology with key immunological concepts and aims to put pharmacological data into the hands of immunologists. Pharmacological databases are a vital resource to researchers through providing accurate information on the fundamental science underlying drug action. This extension to the existing IUPHAR/BPS Guide to Pharmacology supports research into the development of drugs targeted at modulating immune, inflammatory or infectious components of disease.

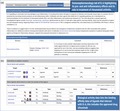

**Abbreviations**
BPS: British Pharmacological Society
CANTOS: Canakinumab Anti‐inflammatory Thrombosis Outcomes Study
CD: cluster of differentiation
CIRT: Cardiovascular Inflammation Reduction Trial
CLL: chronic lymphocytic leukaemia
COX: cyclooxygenase
CRP: C‐reactive protein
CVD: cardiovascular disease
FDA: US Food and Drug Administration
GO: Gene Ontology
GtoImmuPdb: Guide to Immunopharmacology
GtoPdb: Guide to Pharmacology
HLA‐E: human leucocyte antigen E
IL: interleukin
INN: International Nonproprietary Names
IRAK: interleukin‐1 receptor‐associated kinase
IUIS: International Union of Immunological Science
IUPHAR: International Union of Basic and Clinical Pharmacology
MAS: macrophage activation syndrome
MI: myocardial infarction
*NAR*: *Nucleic Acids Research*
NC‐IUPHAR: Nomenclature Committee of the International Union of Basic and Clinical Pharmacology
NIH: National Institutes of Health
NLRP3: NOD‐like receptor family 3
NOD: nucleotide‐binding and oligomerization domain
NSAIDs: non‐steroidal anti‐inflammatory drugs
OMIM: Online Mendelian Inheritance in Man
PCSK9: proprotein convertase subtilisin/kexin type 9
SID: PubChem substance record ID
WHO: World Health Organization

## Introduction

The immune system has become a major target for new therapeutics, with approximately 20% of new drug approvals in the last 5 years targeting elements of the immune system (http://www.fda.gov/drugs/development-approval-process-drugs/new-drugs-fda-cders-new-molecular-entities-and-new-therapeutic-biological-products). A high proportion of diseases are also associated with an immune or inflammatory component or process. In particular, chronic age‐related diseases such as Alzheimer's disease, atherosclerosis and diabetes have inflammatory components.^1^, ^2^, ^3^, ^4^, ^5^ The significant roles that inflammation and immune mechanisms play in cardiovascular disease (CVD) have also made them potential therapeutic targets in its treatment.^6^ Autoimmunity is a serious problem, for example in multiple sclerosis,^7^, ^8^ Sjögren's syndrome,^9^ inflammatory bowel disease^10^, ^11^ and rheumatoid arthritis.^12^ Such conditions may coexist with depressive disorders.^13^ There is also much interest in the use of immune therapies, such as the potential exploitation of dendritic cells, to treat cancer.^14^, ^15^

The International Union of Basic and Clinical Pharmacology (IUPHAR) and the British Pharmacological Society (BPS) collaborate on the development and maintenance of the Guide to Pharmacology (GtoPdb, http://www.guidetopharmacology.org). This database is an expert‐curated resource of ligand‐activity‐target relationships, selected from high‐quality pharmacological and medicinal chemistry literature. It has its origins in IUPHAR‐DB and the BPS Guide to Receptors and Channels, both of which focused on receptors and channels.^16^, ^17^, ^18^ The scope of GtoPdb has expanded over the years^19^, ^20^, ^21^, ^22^ and a Wellcome Trust‐funded project has allowed us to address the priority area of immunity, inflammation and infection.^23^, ^24^, ^25^, ^26^ In the course of that project, the database has expanded into the field of immunopharmacology.^21^

Well‐curated pharmacological databases are an important foundation for research on new therapeutics. In the context of immunopharmacology, although there are good internet resources that support purely immunological research, for example Immunopaedia (http://www.immunopaedia.org.za), ImmPort (http://www.immport.org), ImmGen (http://www.immgen.org), InnateBD (http://www.innatedb.com) and IMGT (http://www.imgt.org), none cover the pharmacology of the immune system. The IUPHAR Guide to Immunopharmacology (GtoImmuPdb; http://www.guidetoimmunopharmacology.org) has been developed to deliver a knowledge‐base that, for the first time, connects immunology with pharmacology.^27^ It expands the data associated with targets and ligands to cover immunological data types and enhances access to the pharmacological data through a user interface tailored to the immunologist. GtoImmuPdb puts valuable pharmacological data into an immunological context and is a resource that enables researchers to easily identify pharmacological agents that can be used experimentally to modulate immune system mechanisms.

### The IUPHAR/BPS Guide to Pharmacology

The Guide to Pharmacology holds data on nearly 3000 human proteins, with over 1700 of these ‘targets’ having curated pharmacological interaction data. In total, the database has information on over 9700 ligands, and it contains quantitative data on over 14 000 ligand–target interactions. The selection of content is supported through the expertise of 96 target family subcommittees of the Nomenclature Committee of IUPHAR (NC‐IUPHAR), comprising over 500 scientists worldwide. GtoPdb uses expert human judgement at all stages of curation, in contrast to more automated data and text mining approaches. Curation is not though limited to only the NC‐IUPHAR subcommittees; we also encourage users to make suggestions about content, which when checked often results in appropriate additions or qualifications.

The GtoPdb is a well‐used and highly cited resource. Our analytics show that the database is accessed by over 22 000 users worldwide each month and they generate a total of more than 118 000 page views. We produce two main biennial publications. The most prominent of these is the Concise GtoPdb,^28^ which provides concise overviews of the key properties of nearly 1800 human drug targets with an emphasis on selective pharmacology. The last two editions (2015/16^29^ and 2017/18^30^) combined have over 2600 citations. We also produce a biennial publication in *Nucleic Acids Research* (*NAR*) Database Issue, which documents database and curatorial updates. Our 2016^20^ and 2018^21^ papers have been cited over 1460 times.

## The IUPHAR Guide to Immunopharmacology: development and curation

In establishing the GtoImmuPdb, NC‐IUPHAR expert subcommittees identified targets relevant to immunopharmacology, and they provided detailed curatorial comments on the reasons for their inclusion in the resource. In the 2019.5 database release, 614 targets and 1232 ligands were tagged as relevant to immunopharmacology (http://www.guidetoimmunopharmacology.org//immuno/immunoHelpPage.jsp#gtoimmupdb_content).

### Curating GtoImmuPdb data

The first phase of curation involved assessing protein targets and ligands that were already in the GtoPdb for inclusion in the GtoImmuPdb. To extend coverage beyond what was already in GtoPdb, we made use of the Gene Ontology (GO) ‘biological process’ annotations to prioritize targets for curation. We produced a draft list of targets for inclusion in GtoImmuPdb on the basis of both direct involvement in inflammation/immunity and based on involvement in processes known to be important in inflammation/immunity. Ligands for targets that qualified for GtoImmuPdb were then reviewed and included if there was evidence that their activity has a modulatory effect on the inflammation/immune system (e.g. drugs approved to treat inflammatory conditions, or tool compounds used to investigate GtoImmuPdb targets). The selection of content for curation was supported by the NC‐IUPHAR subcommittees, who identified key papers and literature reviews. Examples of inclusions identified at this stage are histamine receptors (http://www.guidetoimmunopharmacology.org/GRAC/FamilyDisplayForward?familyId=33)^31^ and anti‐histamine drugs, glucocorticoid receptor (http://www.guidetoimmunopharmacology.org/GRAC/ObjectDisplayForward?objectId=625)^32^ and anti‐inflammatory glucocorticoid drugs, cyclooxygenase enzymes (http://www.guidetoimmunopharmacology.org/GRAC/FamilyDisplayForward?familyId=269)^33^ and non‐steroidal anti‐inflammatory drugs, and pattern recognition, cytokine and chemokine receptor families. Examples of new content added during this phase include additional families of pattern recognition receptors^34^ and immune checkpoint proteins, and ligands and immune checkpoint inhibitors (clinical and investigational) used in immuno‐oncology.

Targets and ligands continue to be added to the GtoImmuPdb as new evidence emerges. Ongoing updates are driven by systematic searches of current literature covering immunology and inflammation to identify lead compounds, their molecular targets and pharmacological data. Other useful resources include pharmaceutical companies' declared development programmes and selective patent analysis that can be used to identify pharmacological data in the absence of peer‐reviewed publications. Review of clinical trial registries, applications to the World Health Organization for new International Nonproprietary Names (INN) (which provide an indication of developments in the immunity/inflammation/immuno‐oncology fields), and monitoring new drug approvals can all identify novel ligands, protein targets and molecular mechanisms of action.

### Immunological processes and cell types

The data on targets and ligands have been extended by annotating these with immunological data. This means we have made clear connections between immunological processes, cell types and disease, and the targets and ligands already in the database. We have made use of biological ontologies because they provide an organized, hierarchical and controlled vocabulary against which to annotate data. Ontologies also provide unique accession numbers that identify a particular term, and these are valuable in supporting interoperability between data resources. In the context of GtoImmuPdb, they are also useful in curating protein targets to different categories and in enabling inferred searching. We have used biological processes from the GO^35^, ^36^ (http://geneontology.org) and cell types from the Cell Ontology^37^ (http://obofoundry.org/ontology/cl.html).

The GO is a hierarchical ontology that describes biological processes, including processes that operate in the immune and inflammatory systems.^38^, ^39^ GtoImmuPdb uses top‐level process categories, such as *T cell (activation)* or *Cytokine production and signalling*, underpinned by GO immune and inflammatory process terms. In the case of T‐cell activation, this includes terms such as ‘T‐cell‐mediated immunity (GO:0002456)’ and ‘regulation of T‐cell differentiation (GO:00045580)’.

The Cell Ontology is designed as a structured vocabulary for cell types, from prokaryotes to mammals. In a similar way, GtoImmuPdb uses top‐level cell type categories, such as *Mast cells*, because of their relevance in anti‐allergic therapies,^40^ and *Innate lymphoid cells*, reflecting the growing understanding of their role within the innate immune system in the control of tissue homeostasis, infection, inflammation, metabolic disease and cancer.^41^, ^42^ The top‐level categories are underpinned by Cell Ontology terms, which in the case of *Mast cells* includes the terms ‘mast cell (CL_0000097)’ and its children, ‘mucosal type mast cell (CL_0000485)’ and ‘connective tissue type mast cell (CL_0000484)’.

Table 1 shows associations between the top‐level processes and the number of human immunopharmacological target proteins. The table also shows the number of human target proteins relevant to immunopharmacology associated with the top‐level cell types. More details of how data have been curated can be found in our recent publication.^21^

**Table 1:** GtoImmuPdb Process and Cell Type categories and the number of human proteins associated with each group

Process	Annotated human targets	Cell type	Annotated human targets
Barrier integrity	49	B cells	51
Inflammation	633	Dendritic cells	41
Antigen presentation	142	Granulocytes	46
T cell (activation)	196	Innate lymphoid cells	6
B cell (activation)	161	Macrophages	56
Immune regulation	503	Mast cells	39
Tissue repair	19	Natural killer cells	26
Immune system development	251	Other T cells	3
Cytokine production and signalling	504	Stromal cells	1
Chemotaxis and migration	256	T cells	76
Cellular signalling	476		

## The IUPHAR Guide to Immunopharmacology: accessing the data

The GtoImmuPdb portal allows researchers with a primarily immunological background to find pathways, drugs and targets using an interface built around an immunological perspective. Immunological processes, cell types, pathways and diseases are centre‐stage and connect to search functions that prioritize immunologically relevant pharmacological data. This provides rapid access to lists of targets and ligands relevant to immunopharmacology or allows the viewing of lists of targets and ligands associated with immunological processes, cell types and diseases. In this way, GtoImmuPdb equips immunologists with a means to discover pharmacological agents that are useful in their research and provides a foundation for developing research into therapeutic modifiers of the immune system.

### Navigating the database from a starting point of immunological process of cell type

**Figure 1:**
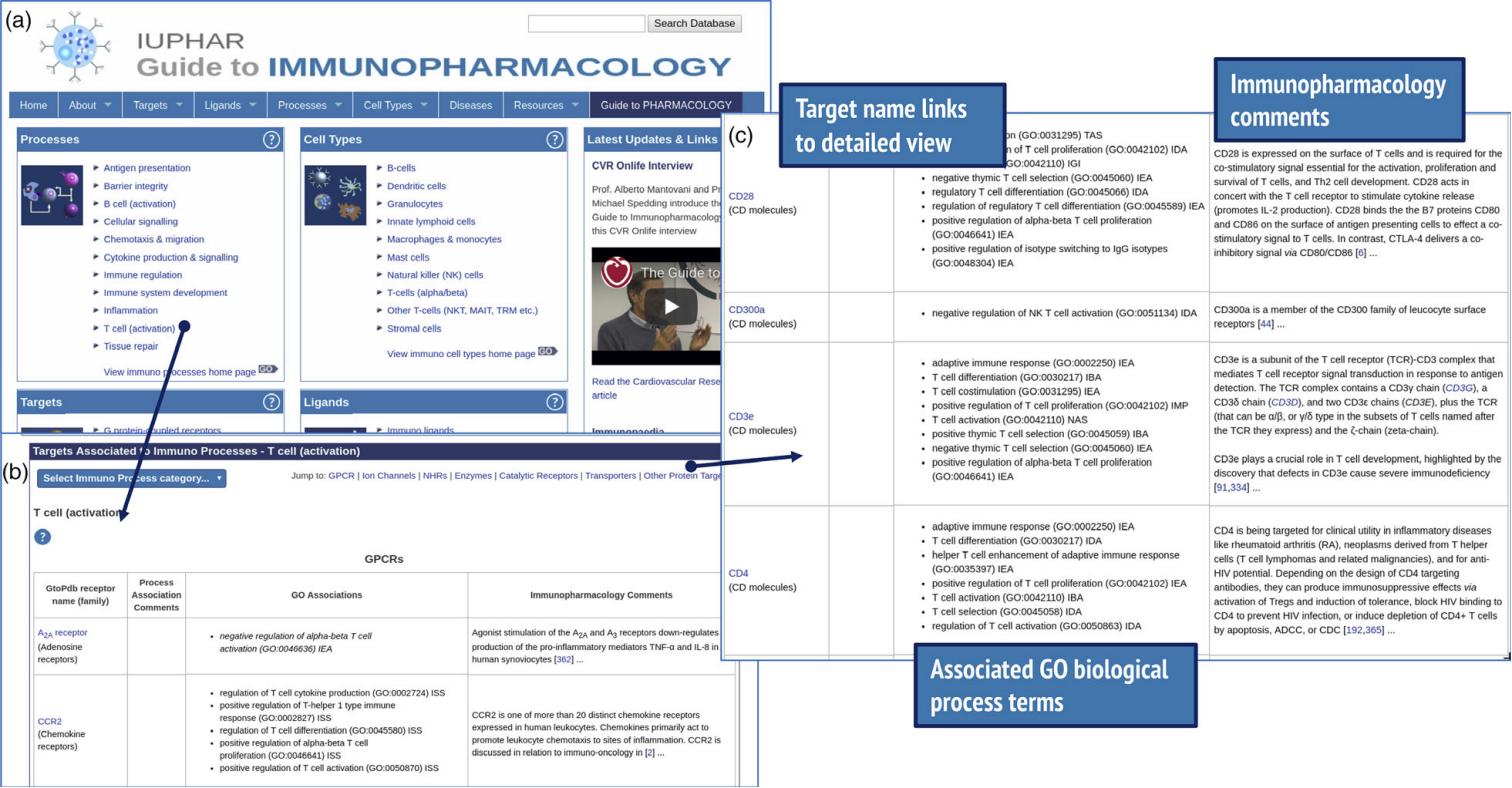
Browsing for targets associated with an immunological process. The GtoImmuPdb portal is shown in (a), with the Processes panel linking to lists of targets associated with T‐cell activation (b). Under the Other Proteins section (c) cluster of differentiation (CD) molecule targets are listed, and in the example of CD28, curatorial comments indicate its role in the activation, proliferation and survival of T cells.

**Figure 2:**
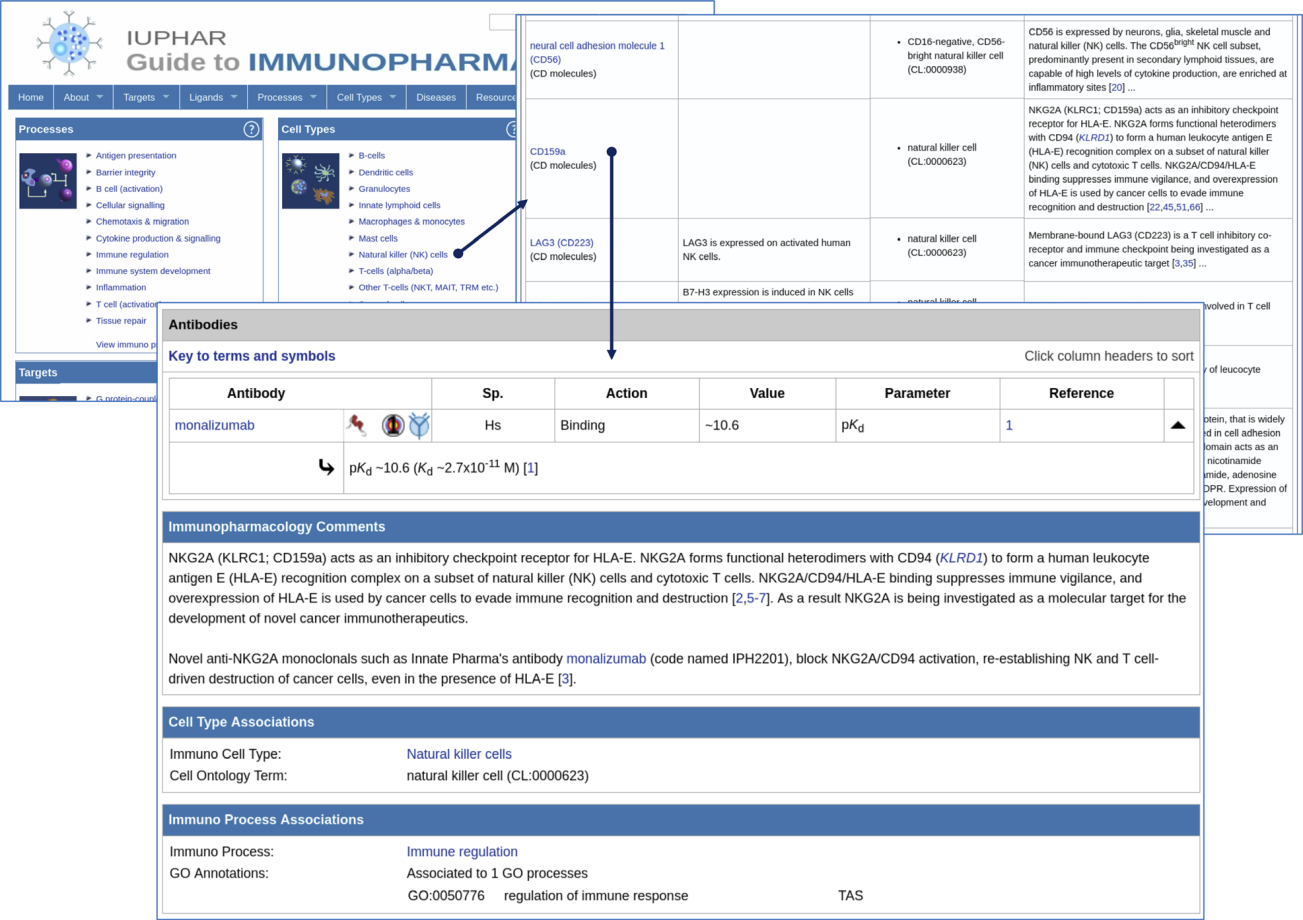
Pharmacological data associated with an immunological cell type. The example shows linking from the portal via the cell type category of Natural killer (NK) cells. The resulting list of targets associated with NK cells includes CD159a. Selecting the link through to the detailed view page shows CD159a interaction with the antibody monalizumab, an anti‐NKG2A clinical lead for haematological cancer.

The database contains nearly 200 targets associated with T‐cell activation (Table 1). These can be easily accessed via the *processes* panel on the GtoImmuPdb portal (Fig. 1a). The targets are organized into sections, one for each target class. Figure 2(b) shows how some cluster of differentiation (CD) molecule targets are displayed in the Other Protein Targets section. The GO terms annotated to a target are shown in the third column of Fig. 1(b); summarized curatorial comments are also displayed. In the example of CD28, its role in the activation, proliferation and survival of T cells is indicated. By clicking on the target name, users can view the detailed targets page, which contains the expanded curators' comments and full pharmacological information on the target.

The annotation of targets to cell types helps to highlight useful pharmacological data relevant to immunopharmacology. For example, the role of natural killer cells in anti‐tumour immunity is well established,^43^, ^44^ and the heterodimer CD94/NKG2A is known to have a role in recognition of the main type of human leucocyte antigen class I molecules and functions as a true checkpoint in natural killer cell activation.^45^ NKG2A (GtoPdb Target 2849; CD159a) is annotated in GtoImmuPdb as being expressed by cells in the natural killer cells category, with the immunopharmacology commentary highlighting its role as an inhibitory checkpoint receptor for human leucocyte antigen E. The detailed view for CD159a (http://www.guidetoimmunopharmacology.org/GRAC/ObjectDisplayForward?objectId=2849#Antibodies) shows interaction data for the antibody monalizumab (http://www.guidetoimmunopharmacology.org/GRAC/LigandDisplayForward?tab=summary%26ligandId=8323), an anti‐NKG2A clinical lead molecule that is being developed for solid and haematological cancers (Fig. 2).

### Ligand summaries

**Figure 3:**
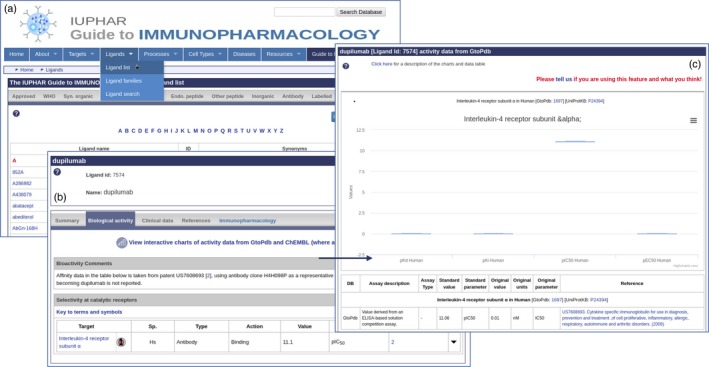
Ligand summary pages. (a) List of ligands is accessed from the menu bar. (b) clicking on a ligand name links to the ligand summary page, here showing dupilumab. Data are presented under several tabs, including one specific to immunopharmacology. Users can link through to the ligand activity visualization tool (c), to compare activities across species.

For ligands, the database contains key information on the biological activity, clinical use, molecular properties, structure and immunopharmacology. These data are displayed on the ligand summary pages, which are easily accessed, either from the Ligands menu bar item, or the Ligands panel on the home page (Fig. 3a). Different categories of ligand can be selected from tabs at the top of the page. When navigating from the GtoImmuPdb portal, the lists contain ligands tagged in the database as relevant to immunopharmacology. Selecting a ligand links through to the ligand summary page where data are organized under several tabs (Fig. 3b). The Immunopharmacology tab contains curator comments on a compound's relevance to immunopharmacology, as well as listing any disease associations. The Summary tab gives general information about the compound, including if the drug is approved for clinical use, and provides a list of trade names (when used clinically), synonyms (such as preclinical names) and INN so that the drug can be identified and tracked in the literature. The ‘Biological Activity’ tab as well as displaying tables of the ligand selectivity at targets in the database also provides access to the Ligand Activity Visualization Tool (Fig. 3c). This tool provides box plots summarizing all the activity data for a ligand taken from ChEMBL^46^ and GtoPdb across multiple species. The Clinical data tab provides information about molecular mechanisms of action and clinical trials together with trial identifying numbers.

### Disease summaries

The extension for immunopharmacology has also prioritized the development of pages that give consolidated pharmacological summaries for different diseases. In all, there are over 1000 diseases in the GtoPdb that have curated associations with targets and/or ligands. The disease lists, accessed from the portal or menu bar, summarize these (http://www.guidetoimmunopharmacology.org/GRAC/DiseaseListForward?type=Immuno). As a consequence of our recent curatorial focus on immunological data, diseases with significant immunological aspects, such as asthma, rheumatoid arthritis, inflammatory bowel and psoriasis, show the greatest number of associations with targets and/or ligands.

**Figure 4:**
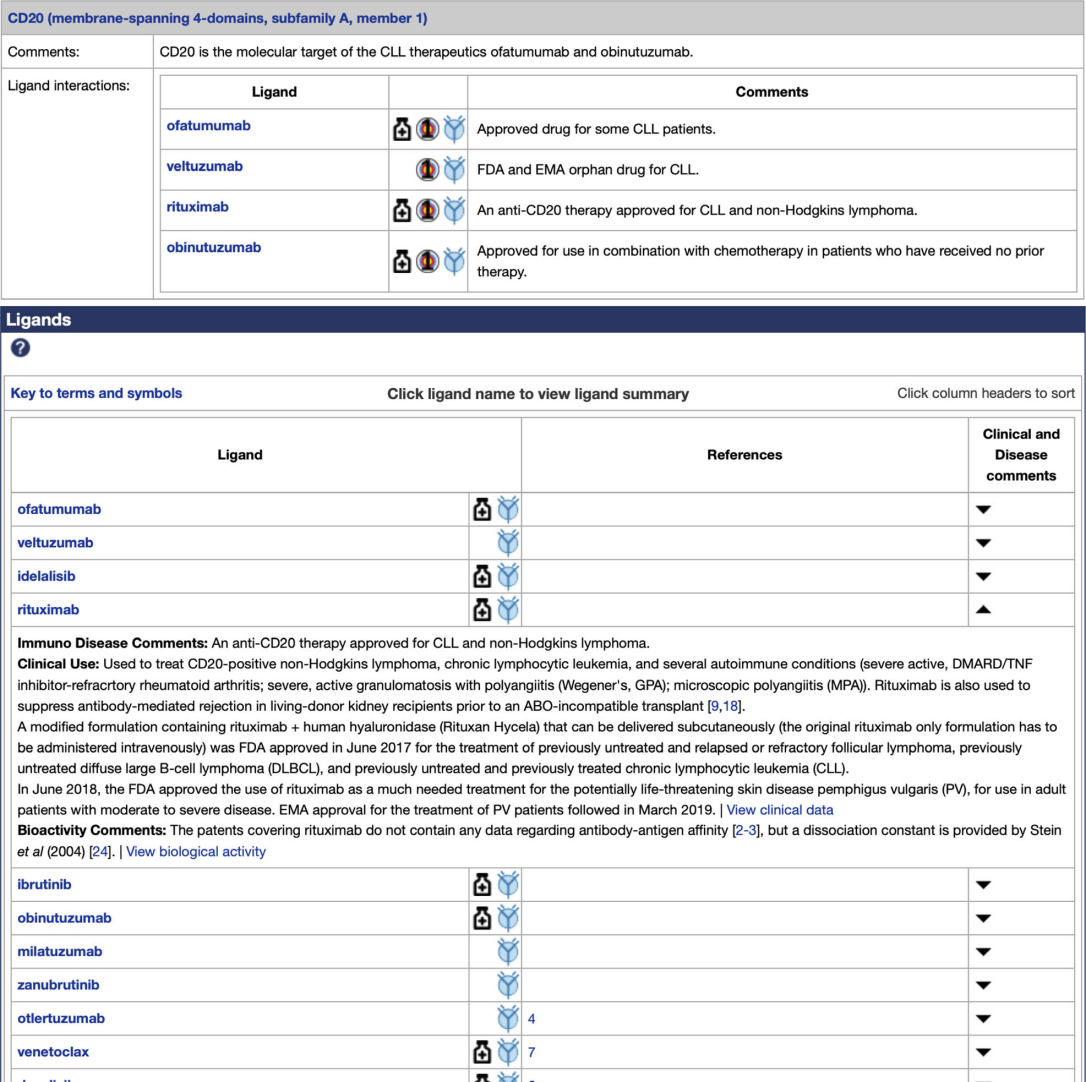
An example disease summary page illustrating chronic lymphocytic leukaemia (CLL; http://www.guidetopharmacology.org/GRAC/DiseaseDisplayForward?diseaseId=218). Four antibodies are highlighted, all of which are therapeutics for CLL, that target CD20. The ligands section provides extended curatorial commentary on the clinical use and bioactivity of the compounds.

The disease summary pages show targets and ligands associated with a disease and include links to Online Mendelian Inheritance in Man^47^, ^48^ (http://omim.org), Orphanet (http://orpha.net) and the Disease Ontology^49^ (http://disease-ontology.org), providing cross‐references between the diseases in GtoImmuPdb and other resources (http://www.guidetoimmunopharmacology.org/GRAC/DiseaseListForward?type=Immuno). The pages also detail the bioactivities and clinical uses of relevant ligands. For example, chronic lymphocytic leukaemia (http://www.guidetopharmacology.org/GRAC/DiseaseDisplayForward?diseaseId=218; Fig. 4) highlights CD20 as being the molecular target of four antibodies: ofatumumab, veltuzumab, rituximab and obinutuzumab. The summarized view shows these antibodies listed against their molecular target and combines this with detailed disease, clinical use and bioactivity comments (Fig. 4). In the case of rituximab, the pages not only explain its role in treating CD20‐positive non‐Hodgkin's lymphoma and chronic lymphocytic leukaemia, but highlight its role in several other autoimmune conditions and in the suppression of antibody‐mediated organ rejection.^50^, ^51^

### Immunopaedia

**Figure 5:**
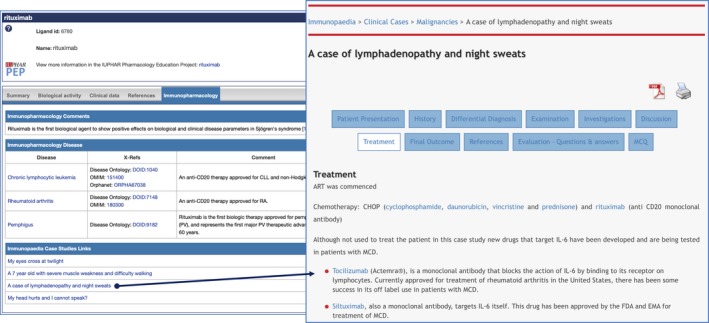
Example of ligand summary page links to relevant Immunopaedia clinical case studies. The illustrated link shows that the antibody rituximab was used in the chemotherapy treatment of a case of lymphadenopathy.

Through the partnership between IUPHAR and the International Union of Immunological Sciences (IUIS) to create standard tools and nomenclature (https://iuis.org/news/2018-iuis-council-meeting-summary/), GtoImmuPdb has been working in collaboration with the IUIS resource, Immunopaedia (http://www.immunopaedia.org.za). Immunopaedia provides materials for teaching and learning immunology, from the basic immune system to advanced immunology and specialized focus areas. They are an official provider for online resources for the IUIS, creating and hosting online courses to educate and support participants before and after immunology conferences worldwide. We have undertaken to provide links from key ligands in GtoImmuPdb to the rich and detailed clinical case studies hosted by Immunopaedia (Fig. 5).

### Searching, web services and PubChem

The search mechanisms across the website have been extended, such that the new immunological data types are incorporated. The search algorithm itself has been tailored so that when using the GtoImmuPdb URL, results of immunological relevance are upweighted. The immunological relevance of a target or ligand is determined by the amount of immunological data associated with it in the database. Our application programming interface has also been extended to incorporate parameters to retrieve immunopharmacology tagged data.

The GtoPdb maintains strong connectivity with PubChem, the open chemistry database at the National Institutes of Health.^52^ On each database release of GtoPdb, we submit our chemical structures to PubChem. As part of this process, we include Depositor Comments in the substance records that we submit to PubChem. These comments, among other things, indicate if a structure is part of GtoImmuPdb and contains any immunopharmacology curatorial comments. Described in more detail in our most recent *NAR* paper,^22^ the inclusion of these comments in our PubChem submissions make it possible to run domain‐specific queries related to immunopharmacology when searching via PubChem.

## Case study: targeting vascular inflammation

The best way to illustrate the potential usefulness of GtoImmuPdb is through a case study. We have chosen vascular inflammation, because in the last three decades, experimental data have clearly shown the causal role played by immune and inflammatory responses in the initiation and development of atherosclerosis, and in the regulation of plaque instability.^6^ Epidemiological studies have also called attention to vascular inflammation. To date, however, there is no immunomodulatory treatment in routine use for prevention of atherosclerosis.^53^ How might GtoImmuPdb help to change this?

The Canakinumab Anti‐inflammatory Thrombosis Outcomes Study (CANTOS; http://clinicaltrials.gov/ct2/show/NCT01327846)^53^, ^54^ has been the first large (>10 000), randomized, double‐blind, placebo‐controlled trial to target the inflammatory cytokine interleukin, interleukin‐1*β* (IL‐1*β*) (http://www.guidetoimmunopharmacology.org/GRAC/LigandDisplayForward?tab=biology%26ligandId=4974) for secondary prevention (to reduce the number of new or severe cases of the disease) of atherosclerosis. In CANTOS, the human monoclonal antibody canakinumab (http://www.guidetoimmunopharmacology.org/GRAC/LigandDisplayForward?tab=immuno%26ligandId=6773) significantly reduced the rate of a composite end‐point of major cardiovascular events in patients previously affected by myocardial infarction (MI) and who had high levels of C‐reactive protein (CRP).^53^ The CANTOS trial represents the first clinical evidence that targeting inflammation may be a viable approach in atherosclerosis and it started an important discussion on how to target vascular immune‐inflammatory responses in the most efficient way (which might not be by targeting IL‐1*β*).

The CANTOS was followed by the Cardiovascular Inflammation Reduction Trial (CIRT; http://clinicaltrials.gov/ct2/show/NCT01594333).^54^, ^55^ In CIRT, treatment with low‐dose methotrexate failed to reduce cardiovascular event rates in patients with previous multi‐vessel coronary artery disease or MI also affected by metabolic syndrome or type 2 diabetes.^55^ It should be noted that despite co‐morbidities, CIRT patients had normal CRP levels and therefore had not been selected on the basis of residual inflammatory risk. Given that high levels of CRP are associated with an increased risk of cardiovascular events, this may help to explain the difference between the CANTOS and CIRT results. In fact, in *post hoc* observations within CANTOS, patients with the largest reduction in IL‐6 and CRP in response to IL‐1*β* inhibition^56^ showed the greatest reduction in cardiovascular mortality, whereas methotrexate had no effect on circulating inflammatory mediators in CIRT. It is worth noting that typing the name of a clinical trial into the main search box on GtoImmuPdb returns a list of ligands involved in the trial, where these data have been curated.

We have learned to a great extent from both trials, but we still have a long way to go before anti‐inflammatory therapies become standard care in the treatment of CVD.^57^

Canakinumab is an expensive agent and it is very unlikely that it will be used in CVD prevention. Several further directions may be investigated, and the first clear opportunity is represented by the targeting of mediators that sit either just above or below IL‐1*β*. More recent analysis from the CANTOS trial revealed that there remains substantial residual inflammatory risk related to both IL‐18 and IL‐6 after IL‐1*β* inhibition.^58^ Therefore, targeting IL‐18 or IL‐6 signalling^59^ could be a way forward. These present us with several questions – can we find a good way to target IL‐18 or IL‐6? Can GtoImmuPdb help in finding a good way to modulate either of these molecules?

### Accessing ligand summaries for IL‐6 and IL‐18

**Figure 6:**
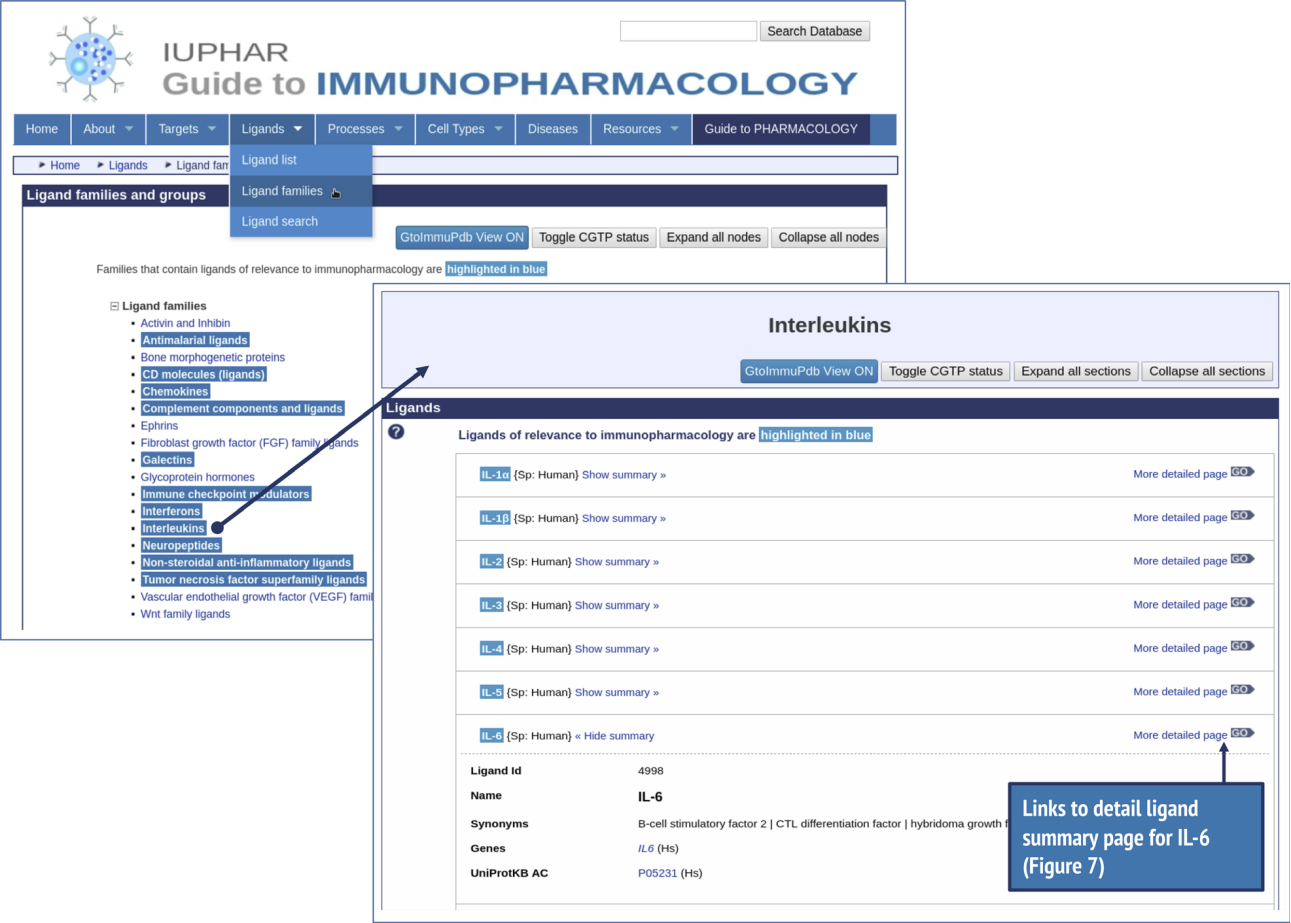
Illustrates accessing ligand summary data using interleukin‐6 (IL‐6) as an example. Browsing via the menu bar for ligand families, selecting the Interleukins group, opens the link through to the Interleukins group. Users can then link through from these points to the IL‐6 ligand summary page (Fig. 7).

To access information about IL‐6 in GtoImmuPdb, go to the portal and type IL‐6 into the database search at the top of any page. IL‐6 is the top‐hit from this search and clicking on the ligand name links through to its ligand summary page. Ligand summary pages can also be accessed by browsing via the Ligand menu bar item, either via Ligand List (alphabetical; Fig. 1), or Ligand Families, which has several groupings of ligands, including one for interleukins, where IL‐6 can be found (Fig. 6).

**Figure 7:**
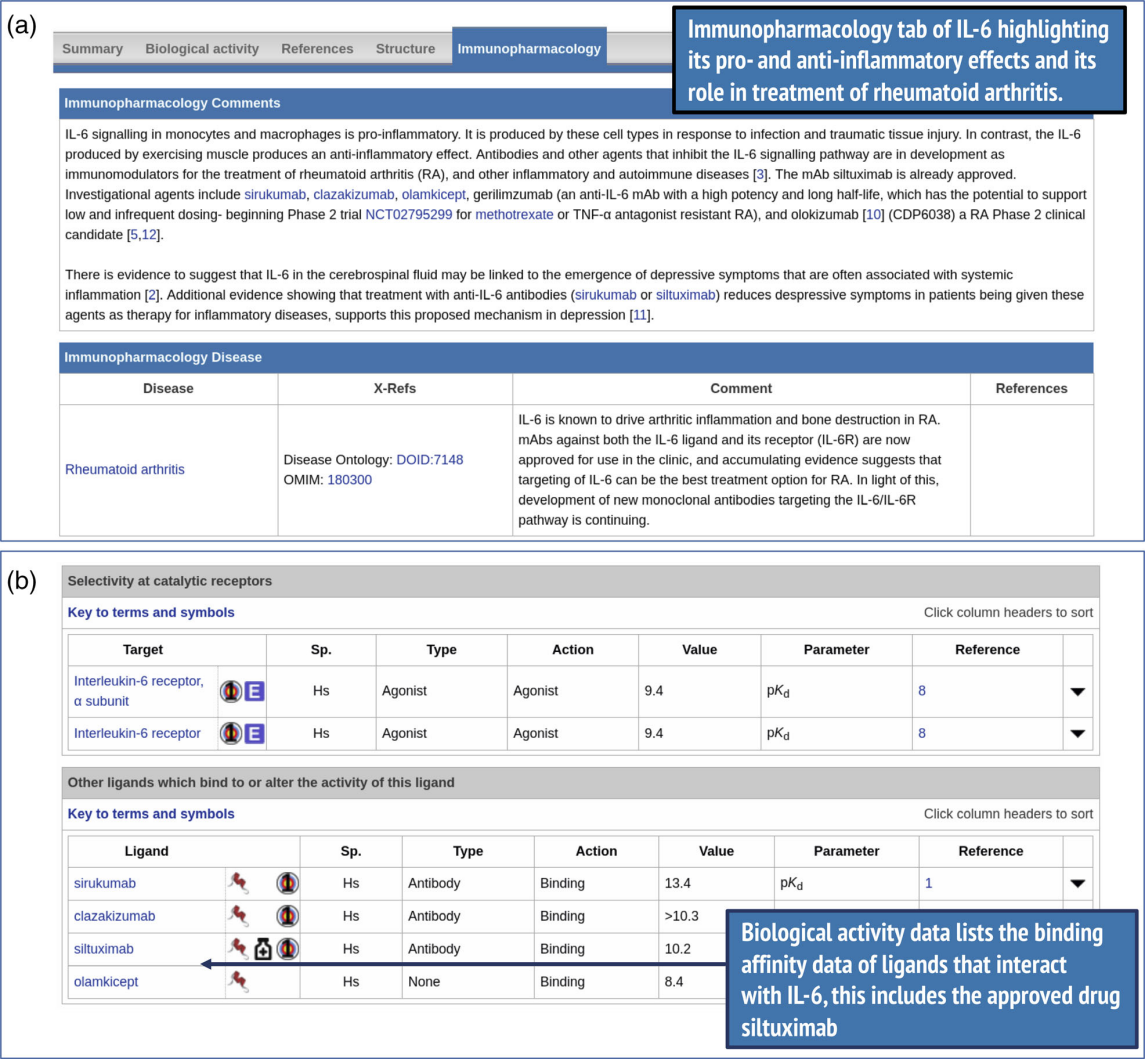
Highlights from the interleukin‐6 (IL‐6) ligand summary page (http://www.guidetoimmunopharmacology.org/GRAC/LigandDisplayForward?ligandId=4998). Curator's comments (a) are shown under the immunopharmacology tab and indicate links with rheumatoid arthritis. Parts of the biological activity tab (b), show ligands that interact with IL‐6, including the approved drug siltuximab.

Information on IL‐6 is contained under several tabs on the ligand summary page (Fig. 7). Figure 7(a) shows information under the immunopharmacology tab, highlighting its pro‐inflammatory and anti‐inflammatory effects and indicating its role in the treatment of rheumatoid arthritis. Figure 7(b) shows biological activity data, which list ligands with which IL‐6 interacts, including binding affinity data and indications of whether the ligands are approved drugs, as is the case for siltuximab. As a starting point when considering a way to potentially target IL‐6, these pharmacological data and immunological context are helpful. They show that IL‐6 is already a validated drug target and a primary target of three ligands including the approved drug siltuximab.

**Figure 8:**
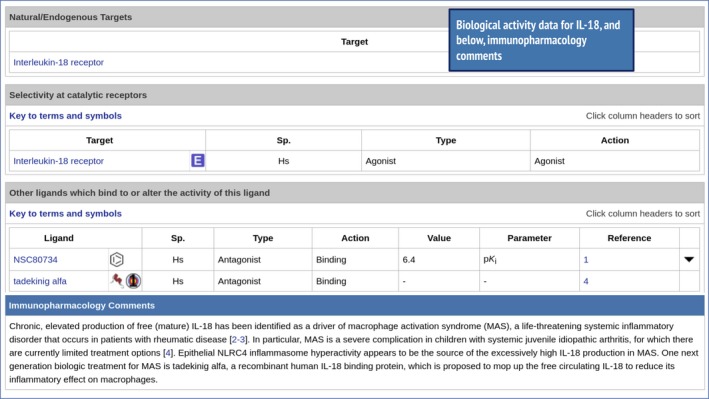
Showing the biological activity and immunopharmacology data from the interleukin‐18 (IL‐18) ligand summary page (http://www.guidetoimmunopharmacology.org/GRAC/LigandDisplayForward?ligandId=4983). Interaction with the tadekinig *α* peptide is highlighted, which plays a role in reducing the inflammatory effect of IL‐18.

Similarly, information on IL‐18 can be accessed in the same way. Figure 8 shows some of the highlights from the IL‐18 ligand summary page, including tadekinig *α*, a peptide ligand that binds to and inhibits the pro‐inflammatory activity of IL‐18 and has US Food and Drug Administration orphan drug designation for the treatment of macrophage activation syndrome. This is useful pharmacological information and context for further investigation of targeting IL‐18.

### Accessing immunopharmacology data for NLRP3 and PCSK9

**Figure 9:**
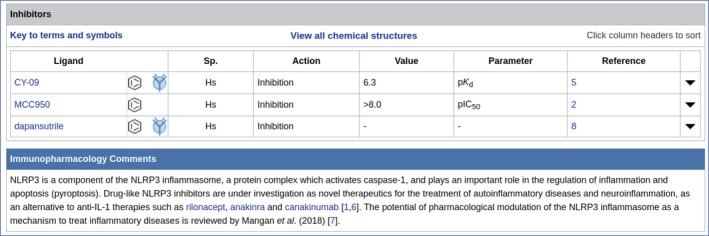
Inhibitors and immunopharmacology data from the detailed target page for NRLP3 (http://www.guidetoimmunopharmacology.org/GRAC/ObjectDisplayForward?objectId=1770#Inhibitors). Immunopharmacology comments highlight its role in the regulation of inflammation. Both CY‐09 and MCC950 have quantitative interaction data and these are marked with the immuno‐icon, showing that they have relevance to immunopharmacology.

Targeting nucleotide‐binding and oligomerization domain (NOD) ‐like receptor family 3 (NLRP3) inflammasome inhibitors that can inhibit both IL‐1*β* and IL‐18^60^ may also present a viable way forward. In this regard, using GtoImmuPdb to view the detailed target page for NLRP3 may be helpful. It is possible to use the direct search to find NLRP3, but it can also be found by browsing through the Catalytic Receptors targets, where NLRP3^34^ is found under the pattern recognition receptors and NOD‐like receptor subfamilies. Figure 9 shows inhibitors and immunopharmacology comments from the NLRP3 detailed target page. Two of the three ligands, CY‐09 and MCC950, have quantitative interaction data for NLRP3 and all three are indicated as having relevance to immunopharmacology. CY‐09 and MCC950 (http://www.guidetoimmunopharmacology.org/GRAC/LigandDisplayForward?tab=immuno%26ligandId=10057#immuno) are shown to have significant therapeutic effects in NLRP3‐driven diseases,^61^ MCC950 (http://www.guidetoimmunopharmacology.org/GRAC/LigandDisplayForward?tab=immuno%26ligandId=8228#immuno) has the potential to block NLRP3‐induced events and there is evidence that dapansutrile (http://www.guidetoimmunopharmacology.org/GRAC/LigandDisplayForward?tab=immuno%26ligandId=10056) is a clinical lead for autoinflammatory disease and heart failure.

**Figure 10:**
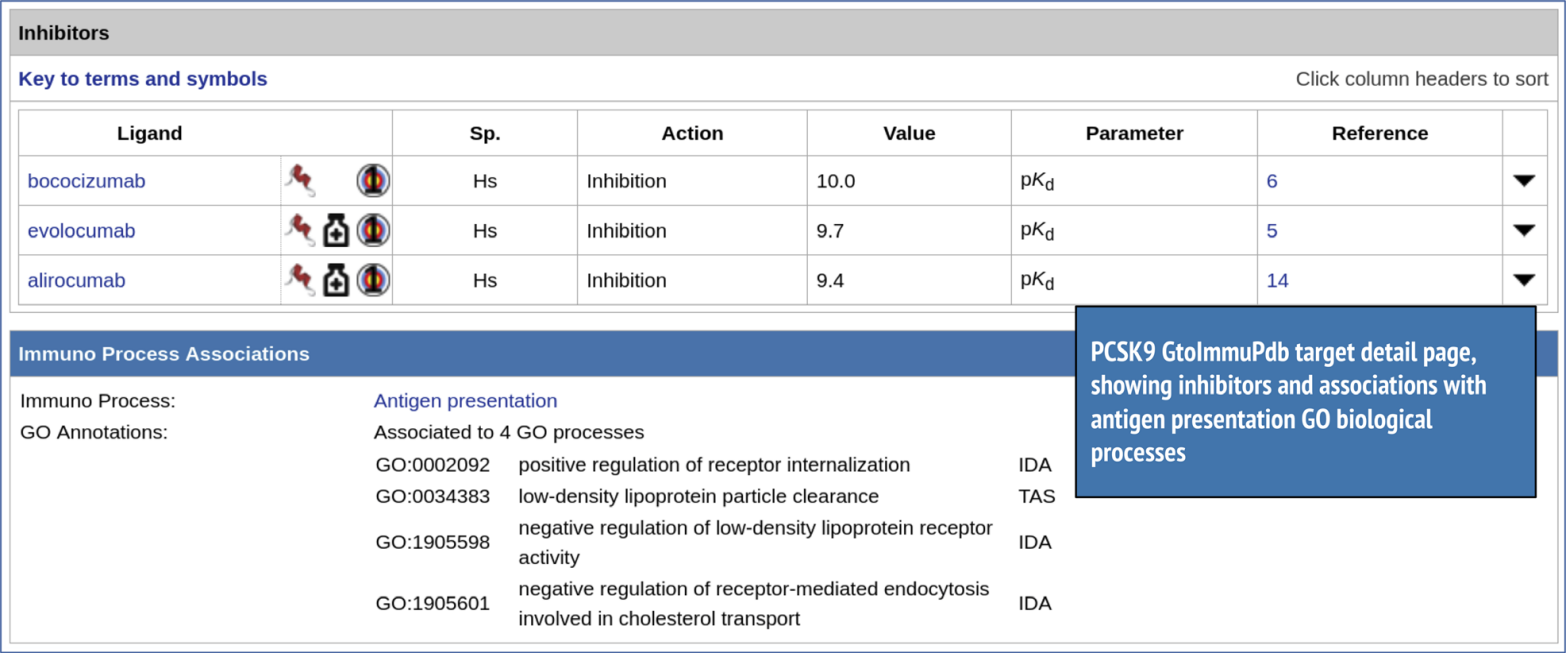
Inhibitors and immunopharmacology data from the detailed target page for PCSK9 (http://www.guidetoimmunopharmacology.org/GRAC/ObjectDisplayForward?objectId=2388#Inhibitors).

A further translational direction may be the development of a novel combination of lipid‐lowering and anti‐inflammatory treatments by design of monoclonal antibodies that could simultaneously inhibit proprotein convertase subtilisin/kexin type 9 (PCSK9; Fig. 10) and either IL‐1*β* or IL‐6. Figure 10 shows inhibitor data from the GtoImmuPdb for PCSK9, showing three monoclonal antibodies with quantitative interaction data for PCSK9. Both evolocumab and alirocumab are approved drugs, and bococizumab is being evaluated in Phase III clinical trials.

Another alternative in targeting the IL‐1*β* pathway could be targeting the IL‐1 receptor itself, or modulating signal transduction downstream of the activated receptors such as members of the IL‐1 receptor‐associated kinase (IRAK) family. In GtoImmuPdb, details for the IL‐1 receptor (http://www.guidetopharmacology.org/GRAC/ObjectDisplayForward?objectId=1905) show that it is already targeted by the antagonist peptide mimic anakira (http://www.guidetopharmacology.org/GRAC/LigandDisplayForward?tab=clinical%26ligandId=6972). For IRAK4, the target detail page shows 11 inhibitors (http://www.guidetopharmacology.org/GRAC/ObjectDisplayForward?objectId=2045%26familyId=579%26familyType=ENZYME#Inhibitors), six of which are selective, including the Pfizer compound (PF‐06650833; http://www.guidetopharmacology.org/GRAC/LigandDisplayForward?ligandId=9667) which is a clinical lead for rheumatoid arthritis, demonstrating that IRAK4 is a druggable target in the pathway.

The testing of new drugs should move in parallel with the identification of better biomarkers for patient stratification, the development of novel molecular imaging modalities for diagnosis and monitoring of vascular inflammation, as well as novel drug‐delivery systems for selective *in situ* targeting of vascular immune pathways and consequent reduced risk of systemic immunosuppression.^62^

## Concluding remarks

The recent appreciation that most chronic diseases include immune aspects, and that modulation of immunity can have a profound effect on disease progression or resolution, makes the immune system a critical target for new therapies. The historically small overlap between immunological and pharmacological research communities has probably hindered the rapid development of immunologically relevant therapeutics. The IUPHAR GtoImmuPdb database and search tools provide a partial solution to this problem, allowing researchers with immunological training to use search terms framed in the concepts of immunology to find pharmacological information and tools relevant to them. In this way, the database should accelerate discovery and development of new strategies against chronic disease.

## Author contribution

SDH designed and developed the database and wrote the manuscript. CS, EF and AP curated the GtoImmuPdb database. PM made a significant contribution to the writing of the manuscript, in particular the case study. SPHA and APD had input on writing the manuscript and as grant holders had roles in the planning of the project. DF contributed to GtoImmuPdb in curation of protein kinases. FLS contributed to GtoImmuPdb in curation of cellular targets, pathways and monoclonal antibodies. MS as grant holder had roles in the planning of the project. JAD contributed to the writing of the manuscript and is the principal investigator of the database development and curation team at the University of Edinburgh.

## Disclosures

There are no conflicts of interest to declare.
